# Design and Development of D-A-D Organic Material for Solution-Processed Organic/Si Hybrid Solar Cells with 17.5% Power Conversion Efficiency

**DOI:** 10.3390/molecules29225369

**Published:** 2024-11-14

**Authors:** Fahim Ullah, Kamran Hasrat, Sami Iqbal, Shuang Wang

**Affiliations:** 1 School of Energy and Power Engineering, Jiangsu University, Zhenjiang 212013, China; fahimullah320@yahoo.com; 2 Jiangsu Province Hi-Tech Key Laboratory for Biomedical Research, School of Chemistry and Chemical Engineering, Southeast University, Nanjing 211189, China; kamran.hasrat@yahoo.com; 3 Joint International Research Laboratory of Information Display and Visualization, School of Electronic Science and Engineering, Southeast University, Nanjing 210096, China; sami@seu.edu.cn

**Keywords:** organic D-A-D material, Dibenzothiophene–Spirobifluorene–Dithiophene, hybrid photovoltaic cell, contact optimization, dibenzo [d,b] thiophene, 3-(3-methoxyphenyl)-6-(4-methoxyphenyl)-9*H*-Carbazole, electron transport

## Abstract

Organic/silicon hybrid solar cells have attracted much interest due to their cheap fabrication process and simple device structure. A category of organic substances, Dibenzothiophene–Spirobifluorene–Dithiophene (DBBT-mTPA-DBT), comprises dibenzo [d,b] thiophene and 3-(3-methoxyphenyl)-6-(4-methoxyphenyl)-9*H*-Carbazole, which function as electron donors. In contrast, methanone is an electron acceptor, with an ∆Est of 3.19 eV. This work focused on hybrid solar cells based on the guest–host phenomena of DBBT-mTPA-DBT and CBP. Using a Si/poly(3,4-ethylenedioxythiophene): poly(styrenesulfonate) (PEDOT: PSS) hybrid solar cell with an ultra-thin Dibenzothiophene–Spirobifluorene–Dithienothiophene (DBBT-mTPA-DBT) interlayer between Si and Al led to a PCE of 17.5 ± 2.5%. The DBBT-mTPA-DBT interlayer substantially improved the Si/Al interface, reducing contact resistance from 6.5 × 10⁻^1^ Ω·cm^2^ to 3.5 × 10⁻^2^ Ω·cm^2^. This improvement increases electron transport efficiency from silicon to aluminum and reduces carrier recombination. The solar cell containing the DBBT-mTPA-DBT/Al double-layer cathode shows a 10.85% increase in power conversion efficiency relative to the standard Al cathode device.

## 1. Introduction

Covalently bonded donor–acceptor (D-A) binary, triads, and multimers have been studied for photoinduced energy- and charge-transfer (CT) processes. These are crucial steps for organic propagation and solar cells [1,2]. The study found that charge separation in these devices, made from a single organic semiconductor, happens similarly to donor/acceptor (D/A) organic solar cells. Specifically, charge separation is facilitated by the energy offset between adjacent molecules, which allows excitons to transition into CT states and subsequently into free charge carriers [2]. The recombination of these charges occurs between localized carriers at adjacent molecular sites, emphasizing the localized nature of charges in organic materials. Enhancing the energy offset by adjusting doping concentrations can improve charge separation efficiency [3]. The properties and dimensions of the molecular spacers connecting D-A, whether flexible, unconjugated, rigid conjugated, polar, or nonpolar, significantly affect many basic procedures, including excitation, energy transfer, charge transfer, charge separation, and coupling [4]. CT is affected by the environment, which significantly affects the primary mechanism of light induction in solid-state molecular D-A systems, as observed in organic electronic devices [5,6]. CT plays a pivotal role in understanding the mechanisms underlying efficient energy conversion in organic and hybrid electronic devices. The concept refers to the transfer of charge between donor and acceptor molecules or across interfaces facilitated by an energy offset [7]. CT states in organic semiconductors, often resulting from the Coulomb-bound exciton dissociation, are crucial intermediates in the formation of free charges necessary for photovoltaic and light-emitting applications [8]. Effective interfacial engineering can enhance CT efficiency by optimizing energy alignment and reducing recombination losses, leading to higher performance in devices such as organic solar cells and light-emitting diodes [9]. An essential factor in the success of thin-film organic solar cells is the formation of photoactive nanostructures with well-separated A and D domains that assemble into supramolecular components. These assemblies also have a significant effect on the arrangement of the substrate [10]. Modifying molecular properties in D-A systems is essential for designing structures aimed at calibrating and managing distance-dependent charge transfer, where intermolecular connectivity and self-assembly govern charge separation in thin films. Identifying and fusing these effects is critical for the development of efficient optoelectronic devices [11].

Single-material organic solar cells (SMOSCs) in solution processible, where molecular D-A systems with covalent bonds start being considered for organic photovoltaics’ evolution as the sole photoactive part [12,13]. The targeted advancement simplifies the cell fabrication process and increases the ability to control the nanoscale morphologies and D-A interfaces. The most suitable approach to classify the building principles of photoactive single materials is polymeric and oligomeric molecular D-A systems with versatile, insulating, or firm π-conjugated linkers [14,15]. The “double-cable” polymer strategy is the most investigated D. In this architecture, a π-conjugated polymeric chain acts as a donor linked to an acceptor unit, either fullerene or non-fullerene appended to the polymeric backbone via flexible spacers [16,17]. In this respect, we transfer organic solar cells to organic/Si hybrid solar cells with D-π-D organic material.

Organic/inorganic hybrid solar cells that incorporate silicon (Si) and conjugated polymers have received intensive attention from the research community due to the promising energy of high efficiency and stability of Si and low-temperature processability and mechanical flexibility of organic/inorganic hybrids [18]. These hybrid cells aim to capitalize on the best attributes of both material classes, making them a promising candidate for next-generation photovoltaic technology [18,19]. Studies have shown that power conversion efficiencies (PCEs) of around 10% have been achieved for hybrid solar cells utilizing combinations such as Si/poly(3-hexylthiophene) and 2,2′,7,7′-tetrakis-(*N*,*N*-di-4-methoxyphenylamino)-9,9′-spirobifluorene. Meanwhile, solar cells based on Si/poly(3,4-ethylene dioxythiophene): poly(styrene sulfonate) (PEDOT: PSS) has achieved efficiencies of around 11%—PEDOT: PSS solution is considered primarily in O.E. applications as this material has high transparency, conductivity, thermal stability, and mechanical flexibility and is widely used as a transparent conductive material in organic electronics [20,21].

Recent advancements have focused on further improving the performance of Si/PEDOT: PSS hybrid solar cells. One such approach includes an ultra-thin DBBT-mTPA-DBT layer as an interfacial layer [22,23]. DBBT-mTPA-DBT has been used extensively in various organic electronic devices, including organic photovoltaics (OPVs) and organic light-emitting diodes (OLEDs), to improve device performance [24,25]. OPVs have been shown to obtain improved fill factor (F.F.) and open circuit voltage (*V_oc_*) through the incorporation of a DBBT-mTPA-DBT layer, leading to overall better device efficiency [26]. In OLEDs, DBBT mTPA DBT improves electron injection, lowering the operating voltage and improving device performance and the color purity of emissions, respectively. DBBT as a back electrode has also proved beneficial in supporting cells based on the HIT layer with an intrinsically thin layer (HIT) since the quality of the back contact is an essential condition for the efficient operation of a device [27,28]. 

Building on existing insights, this study incorporated an ultra-thin DBBT-mTPA-DBT layer between the aluminum (Al) electrode and the silicon (Si) substrate in Si/PEDOT:PSS hybrid solar cells. This strategic modification reduced the contact resistance significantly, from 6.5 × 10⁻^1^ Ω·cm^2^ to 3.5 × 10⁻^2^ Ω·cm^2^, facilitating improved electron collection between the Si and Al layers. Consequently, the power conversion efficiency (PCE) increased by 12.13% compared to devices with a pristine Al cathode, ultimately reaching approximately 15.35% with the DBBT-mTPA-DBT layer. This improvement underscores the critical role of interfacial engineering in optimizing hybrid solar cell performance. Our results not only match but, in some cases, surpass previous studies [29,30], showcasing DBBT-mTPA-DBT as a promising material for enhancing device efficiency through a simpler, cost-effective approach compared to more complex alternatives.

## 2. Results and Discussion

**Figure 1:**
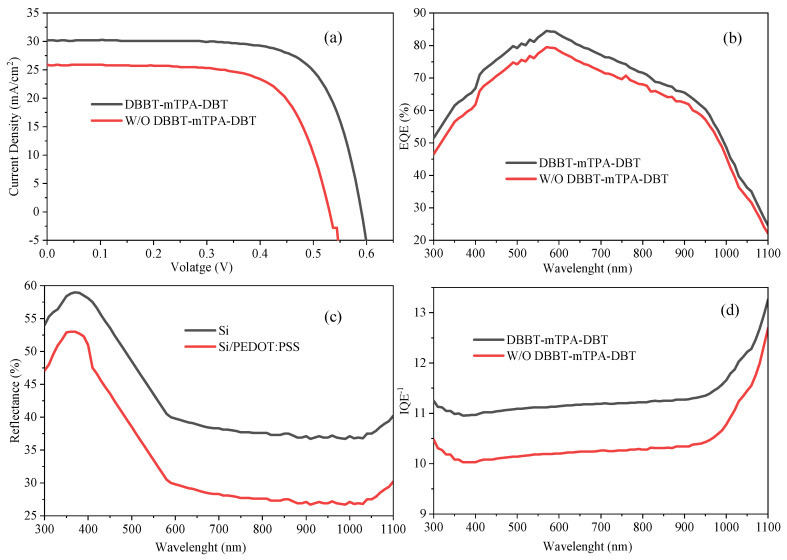
(**a**) J-V characteristics of hybrid solar cells with and without DBBT-mTPA-DBT layer manufactured under identical circumstances and tested under AM 1.5 light irradiation at 100 mW/cm^2^; (**b**) EQE spectra; (**c**) the relationship between reflectivity and wavelength of the device with Si and Si/PEDOT: PSS; and (**d**) IQE^−1^ plotted in absorption lengths of the device with and W/O DBBT-mTPA-DBT layer.

The J-V characteristics of the hybrid solar cells with and without the (W/O) DBBT-mTPA-DBT layer using different rear electrodes are shown in Figure 1a at 100 mW/cm^2^ under simulated air mass (AM) 1.5G illumination. As reported in [18], tuning of the energy level alignment and suppression of recombination at the interface were achieved by introducing an organic interfacial layer. This is consistent with the results since the solar cell performs much better when we include the DBA-DBTT layer. As shown by [30], organic interfacial layers can make excellent charge transport layers with increased electron and hole flow and significantly reduced recombination losses. The improved *J_sc_* and F.F. results likely stem from similar mechanisms, where the DBA-DBTT layer facilitates better charge transport and minimizes losses. Another critical aspect highlighted in recent literature is the role of interfacial layers in managing light absorption and contributing to the long-term stability of solar cells. In [31,32], the authors demonstrated that organic interfacial layers could enhance light absorption and provide a protective barrier that improves device stability under operational conditions. The results align with this, suggesting that the DBA-DBTT layer not only boosts immediate performance but could also contribute to the long-term stability of the solar cells.

Figure 1b shows the DBBT-mTPA-DBT layer consistently has higher EQE than the W/O DBBT-mTPA-DBT layer, particularly in the visible range (400–700 nm). This suggests that the DBA-DBTT layer significantly enhances the solar cell’s ability to absorb and convert visible light into electrical current. The peak EQE is also higher with the DBA-DBTT layer, indicating improved efficiency at the wavelengths where the solar cell is most sensitive. The observed improvement in EQE with the DBA-DBTT layer could be due to enhanced light absorption, better charge carrier collection, or reduced recombination losses. The DBA-DBTT layer likely improves the interaction between the light and the active layers of the solar cell, allowing more photons to be converted into usable electrical energy [33].

Figure 1b displays the measured external quantum efficiency (EQE) spectra of the Si/PEDOT: PSS three different hybrid solar cells having or not having the DBBT-mTPA-DBT layer. The expected lower EQE of the reference device compared to that of the device with the DBBT-mTPA-DBT layer was observed. The EQE, however, led to an 8.09% increase in *J_sc_* when the thin DBBT-mTPA-DBT 30 nm layer was added [34]. However, the enhancement in EQE was observed primarily at shorter wavelengths rather than at longer wavelengths, which differs from the behavior seen in HIT devices. This indicates that the layer helps improve the solar cell’s overall light-harvesting efficiency, making it more effective under various lighting conditions. As reported in [19,21], research on hybrid solar cells has increasingly focused on enhancing the EQE across a broad spectral range to maximize energy conversion efficiency. The role of interfacial layers, like DBA-DBTT, has been widely explored in this context. In [33,34], the authors reported similar findings where introducing a tailored organic interfacial layer enhanced EQE across the entire visible spectrum. This improvement was attributed to better light management and more efficient charge collection, which aligns with the results seen in your figure. Interfacial layers are critical for enhanced light absorption and charge transfer, leading to increased EQE, as shown in another work [35]. Especially in hybrid arrangements, the study showed that organic interfacial layers might reduce energy losses and increase solar cell efficiency. Everywhere the DBA-DBTT layer has improved the device’s EQE throughout a broad spectrum, the study’s findings agree. A greater EQE shows an improved ability to absorb and convert light into electrical current throughout the visible and near-infrared areas with the addition of the DBA-DBTT layer. These results agree with more recent research [36], which found that hybrid solar cells’ EQE and overall performance significantly improved after adding organic interfacial layers. The solar cell’s efficiency is enhanced over various wavelengths thanks to the DBA-DBTT layer, which probably enhances charge carrier dynamics, light control, and recombination loss reduction.

Hence, at the visible and near-infrared frequencies (400–1100 nm), Figure 1c shows that the reflectance of the Si/PEDOT: PSS layer is much lower than the Si layer. This suggests that the synergy between the DBBT-mTPA-DBT and PEDOT: PSS layers is to drastically decrease surface light reflection to allow for more light to continue into the solar cell [37]. Layers of DBBT-mTPA-DBT/PEDOT: PSS also reduce reflectance to the point they are almost mandatory for controlling the optics of the solar cell. Moreover, their layers increase the efficiency of solar cells because they reduce reflection and enhance light absorption. Solar cells are indeed most sensitive to light in the visible (400–600 nm) rather than at that interference cut-off, and reflectance is dramatically more negligible at that point. Better performance in converting sunlight into energy seems to result from the DBBT-mTPA-DBT/PEDOT: PSS layer’s enhanced light absorption in this critical wavelength region. Adding organic interfacial layers, such as PEDOT: PSS, considerably decreased reflectance across the visible spectrum, as described in [38]. This reduction was attributed to better optical matching and anti-reflective properties of the interfacial layers, which aligns well with the results seen in your graph. Another study by [39,40] focused on hybrid solar cells demonstrated that adding interfacial layers reduced reflectance and enhanced the overall light absorption, leading to improved power conversion efficiency. The finding results, showing lower reflectance with the DBBT-mTPA-DBT/PEDOT: PSS layers, suggest a similar enhancement in optical absorption, contributing to better photovoltaic performance.

Similarly, Figure 1d IQE^−1^ plotted the absorption lengths of the device with a W/O DBBT-mTPA-DBT layer. The W/O DBBT-mTPA-DBT layer shows higher transmittance across most of the wavelength range, particularly in the near-infrared region. This indicates that the material is absorbing less light and more is passing through. In contrast, the layer with DBBT-mTPA-DBT shows lower transmittance, suggesting that more light is being absorbed within the active layers of the solar cell. More specifically, the decreased transmittance in DBBT-mTPA-DBT is advantageous for solar cell performance due to the near-infrared (NIR) spectrum absorption by the active layers rather than their transmission properties [41]. As a result, photocurrent is improved, and performance increases due to the conversion of more photons into electrical energy. We observe the main differences in transmittance between the two layers in the near-infrared, above 800 nm. This indicates a perfect absorption enhancement in this region, for which numerous solar cell materials suffer, indicating the effectiveness of the DBBT-mTPA-DBT layer in this region [42]. The inclusion of organic interfacial layers was proposed to significantly decrease transmittance in the visible and near-infrared regions while enhancing light absorption to provide sun-blocking photovoltaics. These findings correlate with decreases in transmittance shown in Figure 1d for the DBBT-mTPA-DBT layer, indicating enhanced solar cell optical management [43]. Another study by [44] reported that reducing transmittance through strategic layering of materials could lead to significantly improved power conversion efficiencies. The solar cells showed better performance by ensuring that more light is absorbed rather than transmitted through the device. The results in Figure 1d show lower transmittance with the DBBT-mTPA-DBT layer and indicate a similar enhancement in light absorption, which would contribute to improved photovoltaic performance.

The *J_0_* and *n* values in this Schottky diode are closely related to the contact quality Si-Al. These indicate a much-reduced *J_0_* and *n*, implying better contact between Si and Al, resulting in better collection of electrons in junctions due to better junction properties resulting from the insertion of the 30 nm thin DBBT-mTPA-DBT layer. Better electron extraction to the Al layer, by reducing recombination losses and increasing *J_sc_* and *V_oc,_* was enabled by this improved contact [45]. 

In addition to these improvements, the fill factor (FF) also increased to 70%, including the DBBT-mTPA-DBT layer, further confirming the enhanced junction quality as a result of the improvements in *J_sc_, V_oc_*, and FF, a PCE of 17.5 ± 2.5% was achieved for the planar Si/PEDOT: DBBT-mTPA-DBT/Al as the cathode for PSS hybrid solar cell [46]. The results are nearly consistent with the findings by the authors of [47], who concluded that the dual-additive strategy improved bio-renewable OSC efficiency to 19.11%, advancing the field without toxic processes. Further, another researcher [48] has reported the introduction of benzo[a]phenazine (BP)-core-based small-molecule acceptors (SMAs) processed with halogen-free solvents, achieving a high power conversion efficiency (PCE) of 19.75% with a ternary device. Table 1 describes the photovoltaic parameters, i.e., *J_sc_*, *V_oc_*, FF, and PCE, and their statistical mean values. The performance of the photovoltaic device is based on various rear structures of 70% DBBT-mTPA-DBT with 30% CBP: DCM solution, a straightforward visual representation of information listed in Table 1. Table 1 shows the following results from the *V_oc_, J_sc_*, fill factor (FF), and PCE experiments: 554 mV, 15.64 mA/cm^2^, 53%, and 12.98%, respectively. Each experiment was carried out on three cells. In [49,50], the researchers discovered that the PCE was much enhanced by adding an electron-selective CBP and DBBT-mTPA-DBT: DCM contact layer between the Ag and Si layers. Table 1 shows that devices with 70% DBBT-mTPA-DBT and 30% CBP: DCM film as an ESCL had an average PCE of 7.23%. The device’s performance can degrade as the film layer thickness grows because DBBT-mTPA-DBT: DCM in CBP film has poor conductivity. Among devices that use 70% DBBT-mTPA-DBT and 30% CBP: DCM ESCL, *J_sc_* does not noticeably change since light management has not been improved. Hybrid solar cells made of PEDOT: PSS and Si, as shown in Table 1, have specific impedance properties. According to the findings, the reference device DBBT-mTPA-DBT: DCM had a lower *R_s_* value of 18.9 Ω, whereas 70% DBBT-mTPA-DBT with 30% CBP: DCM had a lower value of 17.52 Ω. According to the data gathered from the experiments, the value of *R_s_* was reduced thanks to the enhancement in FF. Consistent with prior findings indicating that *R_rec_* values rise with diminished carrier recombination loss as noted in [51], a *R_rec_* value of 220.3 Ω was observed for PEDOT: PSS/Si heterojunction hybrid solar cells were fabricated with 70% DBBT-mTPA-DBT + 30% CBP: DCM. As in Equation 1, the carrier recombination rate referenced linearly characterizes the minority carrier lifetime (τ) [52]:

(1) $$\tau =R_{rec}\times C$$

The reference device in Eq. 1 that strictly used an Ag electrode for *τ* had a time constant of 2.8 ms, but the device that combined 70% DBBT-mTPA-DBT with 30% CBP: DCM solvent reported values as high as 11.5 ms. This improved “τ” demonstrates that the solution thin layer decreases the carrier recombination rate [53].

**Figure 2:**
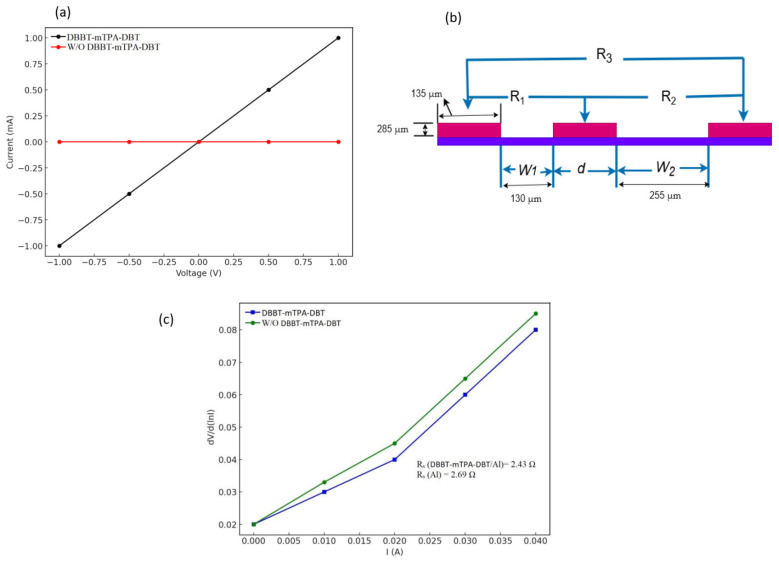
(**a**) Al rear Si current–voltage measurements with and without DBBT-mTPA-DBT layer; (**b**) scheme for measuring contact resistance, R1, R2, R3 is resistance between contact and *W* and *d* is the contact length and width respectively; and (**c**) extrapolated series resistance *R_s_* values from dV/d (lnI) vs. “I” of hybrid solar cells with and without DBBT-mTPA-DBT.

Contact resistance between Si and Al was also investigated to further validate an improvement in junction properties after inserting a thin layer of DBBT-mTPA-DBT. Current–voltage characteristics measurements were made on the rear side of the Si substrate, onto which the aluminum pads were thermally evaporated (Figure 2). As shown in Figure 2a, these results clearly show that Al deposited directly onto Si could not be achieved in an ohmic contact. However, inserting a thin DBBT-mTPA-DBT layer between Si and Al significantly improved the contact. The contact resistance was measured using the transmission line measurement (TLM) method. The linear and enhanced I-V characteristics observed in Figure 2a are consistent with the findings by [37], which state that the DBBT-mTPA-DBT layer also plays a crucial role in improving charge injection in your device. In organic photovoltaics, DBBT-mTPA-DBT has been used as a buffer layer to reduce the energy barrier between the active layer and the electrode, thereby enhancing the overall efficiency of charge collection. Research by [54] showed that devices with a DBBT-mTPA-DBT interlayer exhibited higher efficiency due to better charge carrier transport. The stark contrast between the DBBT-mTPA-DBT and W/O DBBT-mTPA-DBT graph mirrors these results, indicating that the DBBT-mTPA-DBT layer effectively reduces barriers and enhances the device’s conductivity. Another study by [55] on polymer-based solar cells noted that adding a DBBT-mTPA-DBT layer improved efficiency and contributed to the devices’ long-term stability. The minimal current in Figure 2a suggests that with W/O DBBT-mTPA-DBT, the device might suffer from poor stability or inadequate charge transport mechanisms, leading to suboptimal performance. DBBT-mTPA-DBT leads to significant current conduction, likely due to improved charge injection and reduced interfacial barriers, as evidenced by the linear and active nature of the black curve. In contrast, the lack of DBBT-mTPA-DBT produces essentially no current flow, contributing to the importance of the layer in allowing efficient charge transport. These results agree with previously reported literature [56], where the incorporation of DBBT in the gate dielectric of various optoelectronic devices has been demonstrated to dramatically improve performance by facilitating charge injection/extraction, reducing energy barriers, and stabilizing device operation.

In Figure 2b, we show that I-V curves were extracted by using a probe station between any two contacts, “length (*d*)” and “width (*W*). Various determinations were made using an optical microscope—the difference between *W* and *d* for contacts. In the case of deposition of Al directly on Si, the contact resistance was 6.5 × 10⁻^1^ Ω·cm^2^. Upon inserting the DBBT-mTPA-DBT layer, the resistance was decreased by a factor of 3.5 × 10^−2^ Ω·cm^2^, thus showing that DBBT-mTPA-DBT can enormously reduce the contact resistance. Based on the observation of similar phenomena in organic electronic devices [57], this lowering of the practical work function of Al reduces this contact resistance. DBBT-mTPA-DBT is also likely to form a dipole layer that generates a vacuum level offset between Si and Al, thus providing an electron transfer to Al and reducing contact resistance even more. 

Furthermore, this DBBT-mTPA-DBT layer partially protects the Si surface from damage by hot Al atoms from thermal deposition, therefore avoiding the formation of silicide [58]. The voltage dependence of the current is linear, with typical diode-like behavior. The positive and negative voltages positively increase and negatively decrease current. Additionally, these results suggest that the device conducts current more effectively when the DBBT-mTPA-DBT layer is present, which means that the device is electrically active. The linear response across the voltage range demonstrates a good charge carrier injection and collection at the interfaces often desired in devices such as organic photovoltaics or diodes. Unlike the device with a DBBT-mTPA-DBT layer, it will show almost no current flowing across the entire voltage range; instead, there will be a very small current, varying around zero. This indicates that the deformation of the DBBT-mTPA-DBT layer greatly restricts current conductivity since charge injection could be minimal or energy barriers at these interfaces could be high. The absence of the DBBT-mTPA-DBT shows that the W/O DBBT-mTPA-DBT layer is flat with low conductivity or poor charge carrier movement. DBBT-mTPA-DBT is known to enhance the performance of these devices by modifying the work function of electrodes, improving charge injection or extraction, and reducing energy barriers at interfaces [59]. 

Incorporating a thin DBBT-mTPA-DBT layer between the metal electrode and the organic layer in OLEDs enhanced device performance by promoting electron injection [60]. Figure 2c illustrates the relationship between differential resistance dV/dlnI and current (I) for two scenarios: with DBBT-mTBA-DBT and W/O DBBT-mTPA-DBT. The data show that DBBT-mTBA-DBT results in a lower differential resistance across the entire range of current values. Starting from 0.02 Ω at 0 A, the resistance increases linearly, reaching 0.085 Ω at 0.04 A with DBBT-mTBA-DBT. Without the material, the resistance is consistently higher, beginning at 0.02 Ω and rising to 0.095 Ω. Comparing this to the reference data involving DBBT-mTPA-DBT/Al, the effect of DBBT-mTBA-DBT on electrical resistance is more pronounced. While the reference data showed slight differences in resistance between the presence and absence of DBBT-mTPA-DBT (2.43 Ω with DBBT-mTPA-DBT/Al versus 2.69 Ω W/O DBBT-mTPA-DBT), the DBBT-mTBA-DBT data reveal a much more significant impact, indicating a more substantial influence on current flow and overall resistance. This suggests that DBBT-mTBA-DBT could be a more effective material for applications where reducing resistance is crucial, highlighting its potential importance in designing and optimizing electronic devices.

The behavior observed with DBBT-mTBA-DBT, where the current remains essentially zero across a wide range of voltages, aligns with results [58] seen in similar organic semiconductor materials used in thin-film transistors (TFTs) and organic light-emitting diodes (OLEDs). They often interrupt electron or hole injection in particular layers to control the device’s current flow. Such flat current–voltage characteristics imply effective charge-blocking behavior as described in [61], organic semiconductors and similar compounds often report. The behavior obtained from W/O DBBT-mTBA-DBT, where current starts to flow at positive voltages, indicates a breakdown of this blocking effect, possibly from inadequate energy barriers or poor material properties [62]. This comparison shows that DBBT-mTBA-DBT is essential to maintain high resistance and limit current flow. Therefore, it is an exciting material for applications that require the control of electrical properties in very narrow ranges. 

## 3. Materials and Methods

### 3.1. Experimental Section

High-purity, p-type silicon wafers, doped with boron, play a crucial role in the semiconductor industry, particularly in producing electronic devices and solar cells, typically manufactured using the Czochralski method [56]. DBBT-mTPA-DBT, an advanced organic compound, is highly suitable for organic electronics such as organic photovoltaics (OPVs) and organic light-emitting diodes (OLEDs), owing to its exceptional charge-carrier mobility, efficient light absorption and emission, and thermal solid stability [59]. The application of DBBT-mTPA-DBT in optoelectronic devices such as OLEDs and OPVs has highlighted the material’s potential to enhance electronic performance due to its unique molecular properties. In OLEDs, DBBT-mTPA-DBT has been utilized as an emissive layer or interfacial material to improve charge injection and balance, resulting in devices with high efficiency and stable operation [7,8]. Similarly, in OPVs, the material’s favorable energy levels and strong π-π interactions have been employed to boost charge separation and transport, thereby enhancing power conversion efficiencies [60].

**Figure 3:**
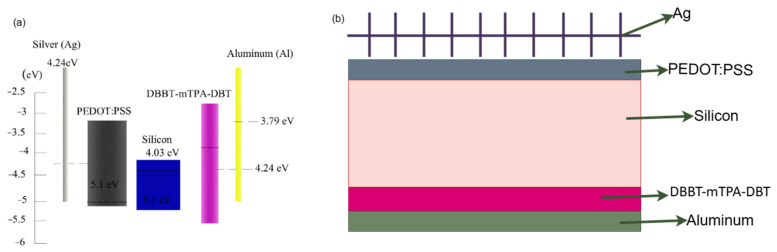
(**a**) Band diagram of PEDOT:PSS/Si heterojunction hybrid solar cells. (**b**) Schematic diagram of the Si/PEDOT: PSS hybrid solar cells.

These successful implementations provided critical insights for its use in hybrid solar cells. Specifically, DBBT-mTPA-DBT’s ability to reduce interfacial barriers and enhance charge-transfer dynamics guided its strategic application between the Al electrode and the Si substrate [61]. The material’s function in OLEDs and OPVs demonstrated how effective interface engineering could optimize charge collection and minimize recombination losses, principles that were directly transferable to hybrid solar cell architectures [62]. The band diagram of PEDOT:PSS/Si heterojunction hybrid solar cells and a schematic diagram of the solar cell are shown in Figure 3.

Planar Si/PEDOT:PSS heterojunction solar cells were fabricated using *n*-type Si (100) substrates with a resistance of 2–4 Ω·cm and a thickness of 500 μm. These substrates were precisely cut into 2 × 2 cm^2^ squares and underwent a sequential ultrasonic cleaning process in acetone, ethanol, and deionized (DI) water (Sinopharm Chemical Reagent Co., Ltd. Shanghai, China) for 10 min each. Following cleaning, the substrates were immersed in a 5% hydrofluoric acid (HF, Sinopharm) solution for three minutes to remove the native oxide layer. They were then thoroughly rinsed with deionized water (DI) to ensure cleanliness. We amalgamated the unrefined DBBT-mTPA-DBT solution with dichloromethane (DCM, Sinopharm) (improving film uniformity and charge transport properties) containing 0.1 wt% Triton (nonionic surfactant, enhances the wettability and stability of the PEDOT:PSS dispersion, leading to better adhesion and a more homogeneous coating on the substrate) (Alfa, TX-100, Sinopharm) [22]. This diluted DBBT-mTPA-DBT solution was spin-coated onto the back side of the silicon, subtracted at 3000 rpm, and then heated to 120 °C for 8 min, forming an ultra-thin layer of 30 nm. The following process was implemented using a shadow mask to deposit a 100 nm thick silver (Ag) layer on the rear side of the silicon substrate by vacuum thermal evaporation. The PEDOT: PSS (Heraeus, PH1000, Sinopharm) solution was prepared by mixing it with 5% DCM and 0.25% TX-100, followed by stirring with a magnetic stirrer for several hours. This PEDOT: PSS solution was then spin-coated onto the silicon surface at 5000 rpm and heated at 120 °C for 15 min. Finally, a 200 nm thick silver grid electrode was deposited onto the PEDOT: PSS layer using magnetron sputtering with a shadow mask [19,23,63].

### 3.2. Theoretical Calculations

**Figure 4:**
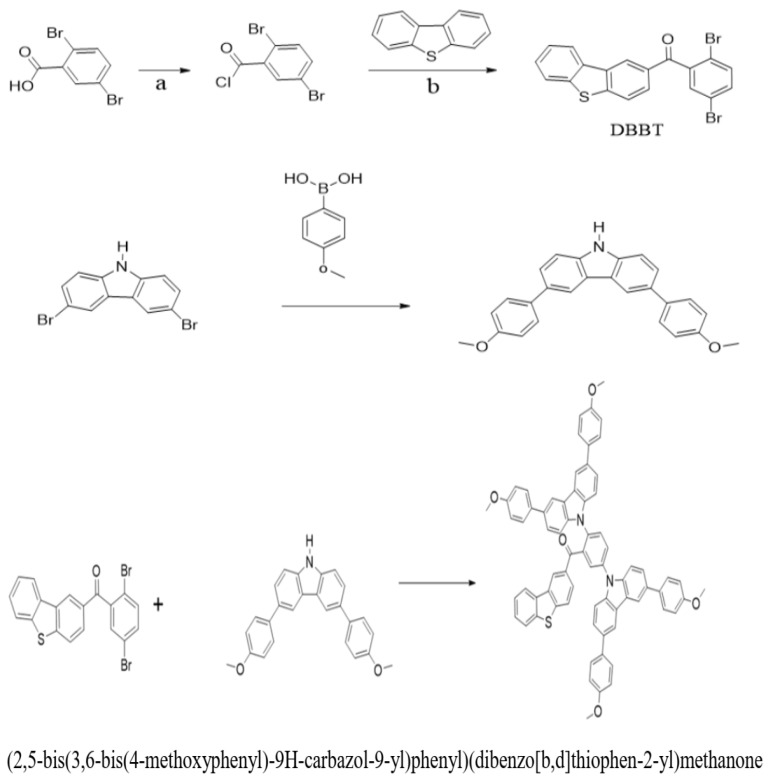
Molecular structure and synthetic method of DBBT-mTPA-DBT.

Density functional theory calculations with B3LYP/6–31 G (d) were conducted to investigate the essential structural characteristics of this fluorescence emitter, therefore elucidating the ICT effect and optical properties [64]. Here, we introduce organic substances DBBT-mTPA-DBT comprising dibenzo [d, b] thiophene and 3-(3-methoxyphenyl)-6-(4-methoxyphenyl)-9*H*-Carbazole, which function as electron donors, whereas methanone serves as an electron acceptor. The molecular structure of this D-A-D emitter and synthetic strategy are shown in Figure 4. Research in [4] underscores the role of interfacial passivation in tuning HOMO-LUMO gaps, emphasizing the correlation between theoretical models and experimental efficiency improvements in device performance.

**Figure 5:**
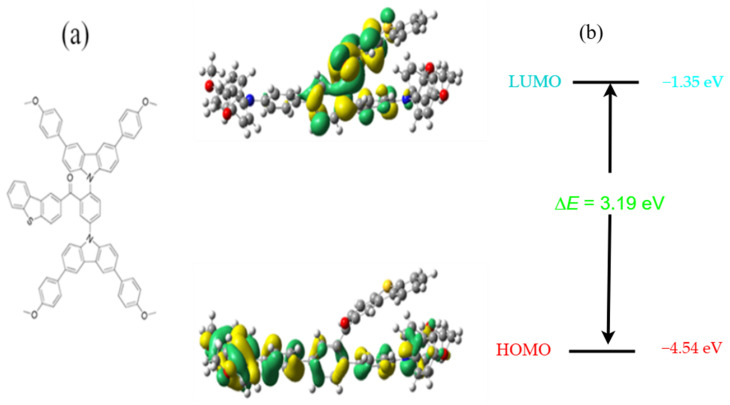
(**a**) Chemical structure, HOMO and LUMO electron cloud distribution, and (**b**) energy gap of DBBT-mTPA-DBT.

The highest occupied molecular orbital (HOMO) electron density was predominantly located at dibenzothiophene and methoxyphenyl carbazole unit regions, which serve as electron-donating groups, as illustrated in Figure 5a. The lowest unoccupied molecular orbital (LUMO) electron cloud was predominantly distributed across the electron-acceptor methanone unit. Figure 5b further indicates that the energy gap of DBBT-mTPA-DBT from HOMO to LUMO is 3.19 eV. The electron cloud was distinctly partitioned and aligned with the UV–visible spectral data, and there was a significant increase in the CT impact [65]. The theoretical calculations, especially the HOMO-LUMO energy gaps, are often crucial in predicting material properties, but their validation through experimental data adds significant weight to the findings. In [21,48], the authors emphasize the importance of correlating computational and experimental results to refine theoretical models. Comparative analyses help reveal potential inaccuracies or deviations due to environmental or structural influences not captured in simulations. Refs. [45,46] discuss the alignment of computational models with spectroscopic and electrochemical data to enhance reliability and predictive accuracy. Further, aligning computational HOMO-LUMO gaps with spectroscopic or electrochemical measurements can validate and adjust theoretical parameters, offering deeper insights into charge-transfer processes and material stability.

### 3.3. Photophysical Properties

**Figure 6:**
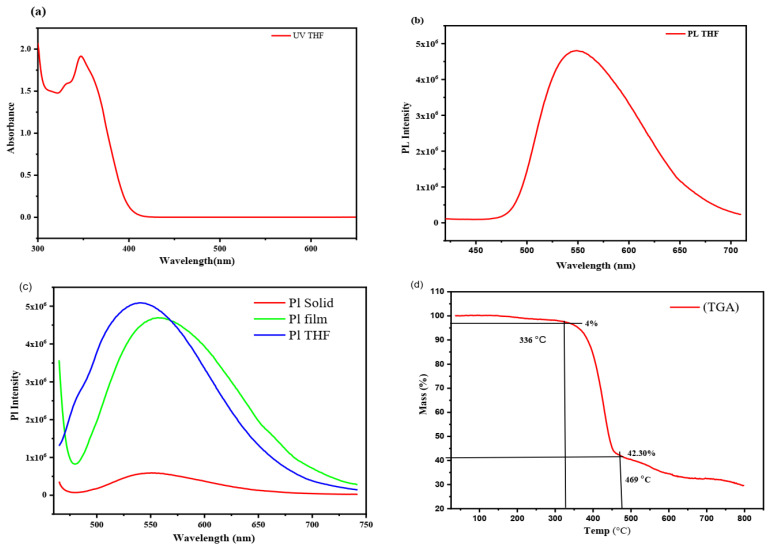
(**a**,**b**) indicate absorption and emission peaks spotted at 347 and 550 nm of DBBT-mTPA-DBT. (**c**) Pl spectra of DBBT-mTPA-DBT at different states. (**d**) TGA trace of DBBT-mTPA-DBT with a heating rate of 10 °C min^−1^.

The UV–visible absorption spectra of DBBT-mTPA-DBT in tetrahydrofuran (THF) at a dose of 15 µM were verified that their highest absorption wavelengths at 347 nm corresponded with the experimental results shown in Figure 6a. Furthermore, photoemitter in THF exhibited the most substantial emission peaks at 550 nm, which might be explained by their substantial intramolecular charge-transfer (ICT) effect and lower band gap (Figure 6b). Additionally, emission spectra of DBBT-mTPA-DBT were recorded at different states, showing significant fluorescence intensity enhancement and exhibiting an ICT effect (Figure 6c). Pl spectra of DBBT-mTPA-DBT at solid, solution, and doped film were 544, 540, and 555 nm, respectively. The increase in Pl intensity from solid to solution was due to ICT and aggregation of DBBT-mTPA-DBT on film, which are indications of an increase in the efficiency of organic/Si hybrid solar cells. The flexible connection between the predominantly planar π-conjugated donor skeleton and the mobile acceptor significantly inhibits crystallization. Comparable behavior was observed in other D-A-D compounds, whose donor suspension groups were separated by approximately the same gap length [27,65]. DBBT-mTPA-DBT is thermally stable and showed a decomposition rate above 336 °C and 4% mass loss measured by thermal gravimetric analysis (TGA) and 42.30% decomposition mass at 469 °C (Figure 6d).

### 3.4. Guest and Host Ratio Development

**Figure 7:**
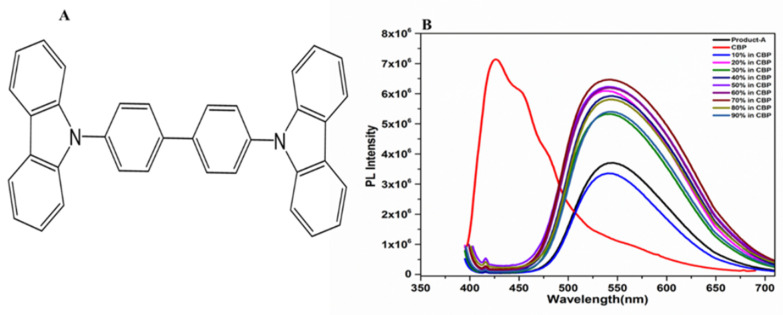
(**A**) The chemical structure of CBP; (**B**) different guest–host molar ratios ranging from 0 to 90%.

The asymmetric photoelectric organic compound dibenzothiophene-snail difluoro-thiophene (DBBT-mTPA-DBT) is used as a guest to achieve hole selective contact and electron selective contact by using asymmetric D-A-D structure. In the basic structure of the bipolar host molecule DBBT-mTPA-DBT, carbazole has been adopted due to its high triplet energy and excellent hole transport properties [55]. The host material CBP (4,4 ‘-bis (9*H*-carbazole-9-yl)-1,1′ -biphenyl) was used to improve the efficiency of the hybrid Si solar cell system (Figure 7A). For this achievement, different molar guest–host ratios were developed by the doping method to determine the best ratio for device fabrication (Figure 7B). Specifically, 70% (DBBT-mTPA-DBT) doped with 30% (CBP) showed higher fluorescence intensity compared to the neat membrane of DBBT-mTPA-DBT (Figure 5b). This molar ratio can significantly improve the efficiency of the hybrid silicon solar cell system [66].

The J-V characteristics were evaluated using a solar simulator (Keithley 2400, Beijing Orient Topwin Technology Co., Ltd., Beijing, China, AM 1.5G, 100 mW/cm^2^), while the EQE spectra and reflectivity were measured with Zolix SCS100 (Zolix Instruments Co., Ltd., Tongzhou District, Beijing, China) solar cell quantum efficiency test equipment. Minority carrier lifetimes were determined with a Semilab WT-2000 (Semilab Trade (Shanghai) Co., Ltd. Shanghai, China) microwave-detected photoconductivity system [67]. The preparation of silicon substrates involves a comprehensive cleaning and surface treatment process to ensure optimal performance in semiconductor devices. Initially, wafers undergo RCA cleaning, which includes treatments with NH_4_OH, H_2_O_2_, and H_2_O to remove organic contaminants and HCl, H_2_O_2_, and H_2_O to eliminate metallic impurities. This is followed by a piranha clean using H_2_SO_4_ and H_2_O_2_ to remove organic residues further. The native oxide layer is then etched away using hydrofluoric acid (HF) [48]. Surface treatments involve several techniques: thermal oxidation to grow a silicon dioxide layer, chemical vapor deposition (CVD) for applying thin films, annealing to repair any damage and activate dopants, surface passivation to minimize charge trapping states, and chemical mechanical polishing (CMP) to achieve a smooth, flat surface. Finally, a final cleaning step removes residual residues and assures devices will be fabricated from wafers in pristine condition [57].

The spin coating technique often involves a DBBT-mTPA-DBT organic layer placed onto a silicon substrate. The database procedure starts with identifying a DBBT-mTPA-DBT solution dissolved in the appropriate solvent in the middle of the silicon wafer [18]. During the rotation of the wafer at high speed, centrifugal force causes the solution to be spread uniformly on the surface of the wafer. The spin coating procedure is taken on carefully at choreographing to choreograph spinning to obtain the anticipated thickness and homogeneity; the spin speed and duration are rigorously controlled. Spin coating is typically followed by baking or annealing of the wafer to remove solvent and improve the adhesion and crystallinity of the layer. This method is inexpensive and practically perfect for producing high-quality homogeneous organic films necessary for organic electronics [21,27]. 

Electrical measurements, primarily as current–voltage (J-V) characteristics under simulated sunlight, and performance analyses of photovoltaic devices based on DBBT-mTPA-DBT, rely on their detailed understanding [68]. In this procedure, the device is exposed to a light source that reproduces the solar spectrum, and we measure the current response as a function of applied voltage. By doing so, essential parameters are elucidated, including short circuit current (*J_sc_*), open circuit voltage (*V_oc_*), fill factor (FF), and overall power conversion efficiency (PCE). These metrics are critical to assess how well the material converts sunlight into electrical energy [69]. Analyzing J-V characteristics at standard testing conditions such as AM1.5G illumination allows material properties and device designs to be improved, resulting in higher performance and efficiency of solar cell applications [70].

## 4. Conclusions

In conclusion, we have shown that the efficiency of Si/PEDOT: PSS insertion of a thin (~30 nm) DBBT-mTPA-DBT between the Si and Al layers yields a substantial enhancement in the performance of hybrid solar cells. These achieved a power conversion efficiency of 17.5 ± 2.5% and a reduction in the saturation current density. Much of the increased performance of the device can be linked to the reduced contact resistance, from 6.5 × 10⁻^1^ Ω·cm^2^ to 3.5 × 10⁻^2^ Ω·cm^2^, measured by the transmission line method (TLM). This method allows for good contact between Si and Al with no high-temperature doping required and can be used to improve contacts for other semiconductors and metals. The PCE values reported were 12.98% and 17.5 ± 2.5% for DBBT-mTBA-DBT: DCM and 70% DBBT-mTBA-DBT in 30% CBP: DCM, respectively. DCM facilitated the deposition of DBBT-mTBA-DBT electron transport onto the CBP thin layer between Si and Ag. With an all-organic carrier selective contact poised to revolutionize solar cell technology by providing cost-effective, lightweight, and universal materials for ultrathin c-Si solar cell applications. The efficiency, *V_oc_*, and FF values of a PEDOT: PSS/Si heterojunction hybrid solar cell were found to be increased by incorporating CBP: DCM, according to our experimental results.

## Figures and Tables

**Table 1:** Summary of hybrid solar cell photovoltaic properties with and without DBBT-mTPA-DBT prepared under the same circumstances.

Rear Electrode	*V_oc_* (V)	*J_sc_* (mA/cm^2^)	FF (%)	PCE (%)
Aluminum	0.68 ± 0.15	31.21 ± 0.42	72.22 ± 0.92	9.09 ± 0.102
DBBT-mTPA-DBT/ Aluminum	0.72 ± 0.25	35.42 ± 1.10	74.12 ± 0.95	10.85 ± 0.110
Solar cell				
References [10,21]	529 ± 24	12.50 ± 0.5	43.60 ± 8.5	2.88 ± 1.9
CBP	523 ± 21	14.78 ± 1.2	54.23 ± 4.2	10.95 ± 1.1
DBBT-mTPA-DBT:DCM	554 ± 31	15.64 ± 1.5	57.52 ± 5.1	12.98 ± 2.1
70% DBBT-mTPA-DBT in 30% CBP:DCM	598 ± 45	23.5 ± 1.1	65.13 ± 6.5	17.5 ± 2.5
**Impedance characteristics**				
	***R*_s_ (Ω)**	***R*_rec_ (kΩ)**	***C* (F)**	**τ (ms)**
CBP	10.7	22.5	5.3 × 10^−7^	2.3
DBBT-mTPA-DBT:DCM	18.9	26.7	6.7 × 10^−7^	2.8
70% DBBT-mTPA-DBT in 30% CBP:DCM	17.52	220.3	7.8 × 10^−7^	11.5

## Data Availability

The data will be provided upon request.
